# The Limits of Test-Based Scrapie Eradication Programs in Goats

**DOI:** 10.1371/journal.pone.0054911

**Published:** 2013-01-23

**Authors:** Fabien Corbière, Cécile Chauvineau-Perrin, Caroline Lacroux, Séverine Lugan, Pierrette Costes, Myriam Thomas, Isabelle Brémaud, Christophe Chartier, Francis Barillet, François Schelcher, Olivier Andréoletti

**Affiliations:** 1 UMR 1225 INRA-ENVT Interactions Hôtes Agents Pathogènes, Ecole Nationale Vétérinaire, Toulouse, France; 2 ANSES, Laboratoire d’études et recherches caprines, Niort, France; 3 INRA, UR 631, Station d’Amélioration Génétique des Animaux, Castanet-Tolosan, France; Creighton University, United States of America

## Abstract

Small ruminant post-mortem testing programs were initially designed for monitoring the prevalence of prion disease. They are now considered as a potential alternative to genetic selection for eradicating/controlling classical scrapie at population level. If such policy should be implemented, its success would be crucially dependent on the efficiency of the surveillance system used to identify infected flocks. In this study, we first determined the performance of post-mortem classical scrapie detection in eight naturally affected goat herds (total n = 1961 animals) according to the age at culling. These results provided us with necessary parameters to estimate, through a Monte Carlo simulation model, the performance of scrapie detection in a commercial population. According to this model, whatever the number of tests performed, post mortem surveillance will have limited success in identifying infected herds. These data support the contention that scrapie eradication programs relying solely on post mortem testing in goats will probably fail. Considering the epidemiological and pathological similarities of scrapie in sheep and goats, the efficiency of scrapie surveillance in both species is likely to be similar.

## Introduction

Transmissible Spongiform Encephalopathies (TSE), or prion diseases, are fatal neurodegenerative disorders occurring in a number of mammal species like sheep and goats (scrapie), cattle (bovine spongiform encephalopathy–BSE), or humans (Creutzfeldt-Jakob disease–CJD). The key event in TSE is the conversion of a normal cellular protein (PrP^c^) into an abnormal isoform (PrP^Sc^) which accumulates in tissues from infected individuals [1]. PrP^Sc^ is currently considered to be the only TSE biochemical marker. According to the prion concept, abnormal PrP would be the causative agent of TSE [2].

Following the BSE epidemics in the UK and the identification of BSE zoonotic properties [3], [4], the control of human and animal exposure to TSE agents has become a priority. A sanitary policy has been implemented based on both eradication of TSE in food producing animals and exclusion of known infectious materials (Specified Risk Materials, SRM) from the food chain. Whereas the SRM retrieval policy is considered to be highly efficient in preventing dietary exposure to cattle BSE, it has a more limited impact on the entry into the food chain of small ruminants’ classical or atypical TSE agents [5].

In 2002, the European Union (EU) implemented an epidemiological surveillance program for TSE in small ruminants, which relies on both passive surveillance and post mortem testing of a fraction of small ruminants at abattoirs or rendering plants (active surveillance). Similar large scale surveillance programs were implemented in several non EU countries, including Canada and the United States of America. This system provides an estimate of TSE prevalence and in the EU it enabled the trend of classical scrapie evolution in populations over the past decade to be monitored [6].

Compared to clinical surveillance, the post mortem TSE screening tests currently carried out on posterior brainstem (obex) clearly improved the monitoring of Prion disease in small ruminants. However, their performances in terms of individual detection are likely to be limited. A large number of studies in TSE infected small ruminants revealed that PrP^Sc^ accumulation in the central nervous system (CNS) occurs late during the incubation phase [7], [8]. Therefore different authors already suggested that lymphoid tissue could be a more rational choice than posterior brainstem (obex) for TSE screening [9]–[11]. In the lack of robust studies that would have compared the relative performance of TSE detection using CNS or lymphoid tissue as targets, it remains impossible to assess the impact of the obex choice on the global performances of the TSE surveillance in small ruminants.

Active surveillance is the main stream for identification of TSE infected flocks/herds and it is now perceived as a possible means for controlling and eradicating TSE in sheep and goat populations. For instance, the United States of America’s animal health authorities have developed the National Scrapie Eradication Program. This program aims, through the post-mortem testing of small ruminants, to eradicate classical scrapie from the US small ruminant population within the next ten years. Of the 3.2 million sheep and 2 million goats that constitute the US adult small ruminant population, 37 192 sheep and goats were tested in 2011 [12], [13]. The potential success of such TSE control/eradication policy is crucially dependent on the capacity of TSE surveillance system to detect infected flocks and herds.

In this study, we first evaluated the performances of TSE detection in goats according to their age and to the targeted tissue. These estimations were based on systematic PrP^Sc^ detection using an EU approved screening TSE test (Biorad TeSeE Sheep/Goat®) and an OIE reference method (immunohistochemistry) in the obex, and a panel of lymphoid tissues, from naturally exposed goats belonging to 8 infected herds.

Through simulation studies we then estimated the capacity of TSE surveillance programs to detect classical scrapie infected herds under different testing scenarios. The model we developed relied on the TSE diagnostic performances we established and on the demographic structure and management practices of the goat population in France. Our results provides a basis for discussing the effectiveness of policies that would rely solely on TSE testing for controlling and eradicating classical scrapie in small ruminants.

## Materials and Methods

### Herds

Over a 3 year period (2002–2005), eight classical scrapie affected dairy goat herds (population size 93 to 390 animals) in which scrapie had been identified through the active surveillance program at rendering plants, were investigated (Table 1). In accordance with the EU 999/2001 regulation (http://eur-lex.europa.eu/LexUriServ/LexUriServ.do?uri=CONSLEG:2001R0999∶20110318:EN:PDF) all the animals in these herds were subsequently culled and destroyed. At the time of this stamping-out the posterior brainstem (obex), the palatine tonsils, the ileal mesenteric lymph node (MLN) and the distal ileum were collected from 98.3% of individuals. Half of each tissue sample was formalin fixed and the other half was frozen at −20°C. Euthanasia and collection of tissues samples at rendering plants were performed in compliance with institutional and French national guidelines, in accordance with the European Community Council Directive 86/609/EEC (http://ec.europa. eu/food/fs/aw/aw_legislation/scientific/86-609-eec_en.pdf). The experimental protocol was approved by the INRA Toulouse/ENVT ethics committee.

**Table 1:** PrP^sc^ detection in posterior brainstem and lymphoid tissue of goats from 8 classical scrapie infected herds.

Herd	Herd size	Official screening test	No. PrP^Sc^ positive individuals/tested individuals*					
		No. positive*/tested	Method	Posterior brainstem	Tonsil	MLN**	Ileum	Overall
**A**	291	2/285	**IHC**	1/285	1/250	0/250	NA	2/250
			**ELISA**	2/285	1/250	1/250	NA	3/285
**B**	93	0/93	**IHC**	0/93	0/93	0/93	0/93	0/93
			**ELISA**	0/93	0/93	0/93	0/93	0/93
**C**	290	17/271	**IHC**	18/162	31/161	31/162	28/161	32/162
			**ELISA**	18/162	36/189	37/290	28/162	38/290
**D**	390	0/375	**IHC**	0/375	0/369	0/375	0/375	0/375
			**ELISA**	0/375	0/369	0/375	0/375	0/375
**E**	162	16/162	**IHC**	22/162	34/161	34/161	28/162	36/162
			**ELISA**	22/161	35/161	36/161	29/162	37/162
**F**	247	16/241	**IHC**	26/245	38/244	38/244	35/235	38/245
			**ELISA**	24/244	40/242	39/238	39/242	42/245
**G**	313	0/306	**IHC**	0/306	0/306	0/306	0/306	0/306
			**ELISA**	0/306	0/306	0/306	0/306	0/306
**H**	208	27/205	**IHC**	35/202	58/203	56/197	47/200	60/205
			**ELISA**	34/197	57/201	58/204	47/202	62/205
**Overall**	1994	78/1938	**IHC**	101/1795	162/1787	159/1788	138/1532	167/1798
			**ELISA**	100/1823	169/1811	171/1917	143/1542	182/1961

* excluding the index case.

** Mesenteric lymph node. Official screening tests on posterior brain stem were only performed in animals older than 18 months by a state accredited laboratory. In parallel, PrP^Sc^ detection by immunohistochemistry and ELISA (Biorad TeSeE sheep/goat®,) was performed in our premises. In some cases samples were not tested because they were either missing or of insufficient amount.

### PrPSc Detection

The posterior brainstem (obex) was first tested by accredited Official Veterinary Laboratories that used either the Biorad TeSeE®, Biorad TeSeE Sheep/Goat® or the Prionics Check Western Test® which are all validated by the EU for TSE rapid screening in small ruminants. These tests performed by accredited Official Veterinary Laboratories will thereafter be referred as “official rapid tests” In parallel, ELISA PrP^Sc^ detection test was carried out on frozen tissues (including posterior brainstem) using Biorad TeSeE sheep/goat® kit in UMR INRA ENVT premises. Tests were performed according to the manufacturers’ instructions. According to regulation, official rapid tests were performed only in animals older than 18 month. In some cases the official rapid tests had consumed all the appropriate material so that it was not possible to apply the Biorad TeSeE sheep/goat® test to obex in our premises.

In parallel, PrP^Sc^ detection was performed on formalin fixed tissues by immunohistochemistry as previously described [14]. Briefly, PrP^Sc^ IHC detection was performed using 8G8 antibody raised against human recombinant PrP protein and specifically recognising the 95–108 amino acid sequence (SQWNKP) of the PrP protein. For each sample a negative serum control was included, in which the primary antibody was either omitted or replaced by purified mouse IgG2a serum.

### Evaluation of Diagnostic Performances

The agreement between IHC and ELISA results obtained in our premises was evaluated by the Kappa coefficient of concordance. Then, under the assumption that an animal was scrapie positive whenever one of its tissues was PrP^Sc^ positive (with either IHC or ELISA), the sensitivity of each test was computed as the ratio of positive results over the total number of true positives. The performances of the official rapid tests, performed by state accredited testing laboratories were also considered in the analysis. No distinction was made between the different tests used (i.e. Biorad TeSeE®, Biorad TeSeE Sheep/Goat® or Prionics Check Western Test®). Confidence intervals (95% level) were calculated using the exact binomial distribution and the sensitivities of the different tests were compared using the McNemar Chi squared test for paired samples. Analyses were also performed at the herd level and within-herd sensitivities were compared using the Fischer exact test. Finally, the sensitivity of the diagnosis obtained when testing tissue was estimated according to three age groups (12 to 24 months old, 24 to 36 months old and over 36 months old). The one-tailed exact Cochran-Armitage test was used to look for an increasing sensitivity with age, and the Fisher exact test was used for paired comparisons between age groups.

### Performance of the Active Surveillance System at Population Level

A Monte Carlo simulation study was carried out to assess the impact of tests’ sensitivities on the global performances of active surveillance programs. The model is presented in detail as supplementary data (Additional Text S1).

The model was based on the structure of the French goat population (herd size – within herd age structure, see additional Figures S1 and S2) as provided by the French ministry of agriculture records (AGRESTE 2007) and follows the general management policy (replacement rate) usually applied in dairy herds (over 90% of the French goat population). The diagnostic performances of TSE detection tests estimated in the first part of our study were used as input parameters. Two different diagnosis options were evaluated, one based on the rapid testing of obex (“official rapid tests”, accredited laboratories), the other using ELISA PrP^Sc^ detection on palatine tonsils (Biorad TeSeE sheep/goat®, our premises).

Two different testing scenarios were considered. In the first one, 100% of slaughtered and found-dead goats older than 2 years were tested. In the other one, 10,000 animals were randomly tested in each surveillance stream (healthy slaughtered and fallen stock), fulfilling the current EU TSE testing requirements for the French goat population (http://eur-lex.europa.eu/LexUriServ/LexUriServ.do?uri=CONSLEG:2001R0999∶20110318:EN:PDF). An infected herd was considered to be identified when a single individual was found positive. For each surveillance scenario, the proportion of scrapie infected herds detected by the surveillance system was estimated.

## Results

### Scrapie Prevalence in Affected Herds and ELISA/IHC PrP^Sc^ Detection Performance

From the eight studied herds a total of 183 out of 1 961 goats (9.33%) were found PrP^Sc^ positive in at least one tissue (posterior brainstem, tonsil, mesenteric lymph node or ileum) (Table 1). There was a huge variation of disease prevalence between the herds; in some herds (B, D and G) no positive individual was identified apart from the index case whereas in one other (herd H) 30.8% of the goats were found positive. Such variability is consistent with observations reported by different authors in sheep flocks [15]–[17].

PrP^Sc^ detection results as obtained in our premises by IHC and ELISA (Biorad TeSeE sheep/goat®) were not significantly different. Only 19 out of the 6,875 paired results were divergent (Table 2), yielding very high Kappa coefficients of concordance between tests (> = 0.98). The nature of the tissue, the herd and the age at testing had no significant influence on the concordance of results. These observations support the contention that both ELISA (PrP^Sc^ rapid detection test) and IHC (as an OIE validated confirmatory method), display similar performances for PrP^Sc^ detection in the posterior brainstem and lymphoid tissues from classical scrapie infected small ruminants.

**Table 2:** Concordance between Immunohistochemistry and ELISA (Biorad TeSeE sheep/goat®) PrP^Sc^ detection results in tissues from classical scrapie infected goats.

Tissue	Number of paired analysis	Number of discordant	IHC positive ELISA negative	IHC negative ELISA positive	Kappa Coefficient (95% CI)
**Tonsil**	1781	4	1	3	0.98 (0.97–1.00)
**Posterior brainstem**	1785	2	2	0	0.99 (0.97–1.00)
**MLN** *	1780	9	3	6	0.97 (0.95–0.99)
**Ileum**	1529	4	0	4	0.98 (0.97–1.00)
**Overall**	6875	19	6	13	

* Mesenteric lymph node.

### Impact of the Age and Tested Tissue on Diagnostic Performances

Using the same dataset, we then determined the relative ability of each tissue (posterior brainstem, tonsil, mesenteric lymph node or ileum) to provide a reliable diagnosis when tested for PrP^Sc^ presence (ELISA, Biorad TeSeE sheep/goat®).

When considering the tested population as a whole (no age stratification) palatine tonsils or MLN provided a detection sensitivity that exceeded 95%. Significantly lower sensitivities (p<0.001) were observed when using the ileum or the obex (Table 3). Curiously the TSE detection tests carried out on posterior brainstem by state accredited laboratories (“official rapid tests”) displayed a lower sensitivity (Se = 47.3%; 95% CI = 39.5–55.18) than both the ELISA and the IHC that were applied in the UMR INRA ENVT laboratory (Se = 58.4% and 57.8% respectively). Whichever tissue was considered, no significant difference was observed between herds (all p-values >0.2).

**Table 3:** Classical scrapie diagnostic sensitivity per age category in infected goats.

			Asymptomatic cases†						
	All cases		12 to 24 months		24 to 36 months		above 36 months		
	(n = 180)		(n = 25)		(n = 49)		(n = 64)		
Tissue	Se (%)	(95% CI)	Se (%)	(95% CI)	Se (%)	(95% CI)	Se (%)	(95% CI)	p**
**Official rapid tests**	47.3^d^	(39.5–55.2)	0.00^a^	(0.0–10.5)	24.5^b^	(13.3–38.9)	43.6^c^	(30.3–57.7)	<0.001
**Posterior brainstem**	57.8^c^	(50.1–65.3)	8.0^a^	(1.0–26.0)	54.2^b^	(39.2–68.6)	61.1^b^	(48.8–73.8)	<0.001
**Tonsil**	94.9^a^	(90.6–97.7)	91.3^a^	(72.0–98.9)	89.8^a^	(77.8–96.6)	98.4^b^	(91.6–99.9)	0.0126
**MLN** *	95.0^a^	(90.7–97.7)	92.0^a^	(74.0–99.0)	95.9^a^	(86.0–99.5)	93.7^a^	(84.8–98.3)	0.2674
**Ileum**	83.1^b^	(76.7–88.4)	75.0^a,b^	(53.3–90.2)	69.4^a^	(54.6–81.7)	88.9^b^	(78.7–95.4)	0.0293

* Mesenteric lymph node.

** Exact one-tailed Cochran-Armitage trend test.

† For official rapid test on obex the analysis included 121 positive cases (17 less than 2 years, 49 between 2 and 3 years and 55 aged 3 years or more).

For each tissue and age category the estimated sensitivity of the diagnostic (Se) and the 95% exact binomial confidence interval (95% CI) of PrP^Sc^ detection (assessed using ELISA Biorad TeSeE sheep/goat® results) was computed. The same computation was also done for official rapid tests as performed by state accredited laboratories on obex. Computation relies on data presented in table 1 and assumed that an animal displaying PrP^Sc^ accumulation in any of its tissue is scrapie infected.

In all cases, percentages with different letters are significantly different (exact McNemar test p<0.05). For asymptomatic cases, within each row, percentages with different letters are significantly different (Fisher exact test p<0.05).

In the four herds that displayed the highest scrapie prevalence, age records were available for 176 out of the 180 PrP^Sc^ positive individuals. Using the asymptomatic but infected goats sub population (138 goats) we estimated the impact of the age at culling and the nature of tested tissue on the diagnostic sensitivity (Table 3).

Using PrP^Sc^ detection in mesenteric lymph node, the diagnostic performances were not statistically different between age groups (p = 0.2674). With tonsil (p = 0.0107) and ileum (p = 0.0354), a slight but significant increase in the diagnostic sensitivity was observed with increasing age of infected individuals. In contrast the sensitivity of detection using posterior brainstem appeared to be strongly dependent on the age of tested individuals (p<0.001). For instance the official rapid tests failed to identify any of the 17 scrapie incubating goats younger than 2 years old and detected only 36 out of 104 asymptomatic positive cases (34.61%) older than 2 years old. However it identified accurately 31 out of the 32 infected individuals belonging to the clinical suspect sub population (96.8%).

These data confirm that the tissue on which PrP^Sc^ detection is carried out, and the age of the tested individuals, strongly influence the performance of diagnostic methods in scrapie infected small ruminants. Beyond this, they also provided quantitative parameters to estimate the performances of scrapie active surveillance at population level.

### Performance of the Active Surveillance System at Population Level

**Figure 1:**
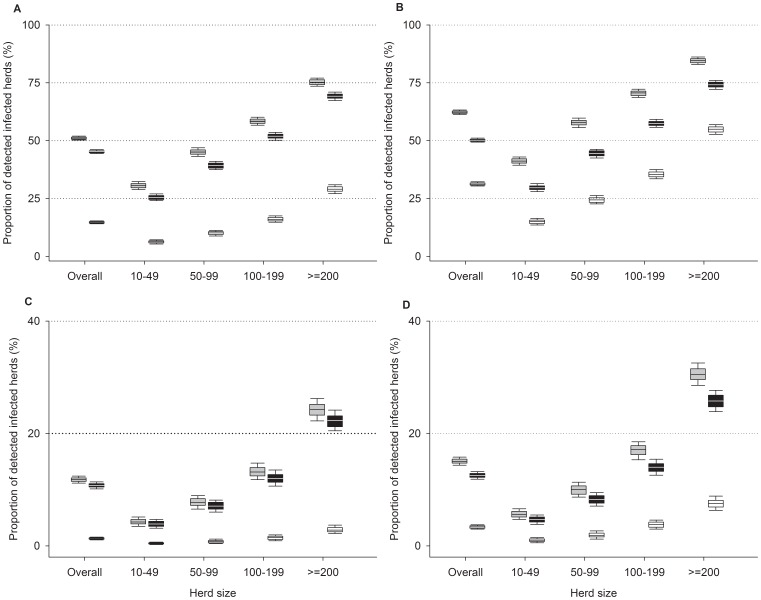
Estimated detection sensitivity of scrapie infected herds in a commercial population through *post-mortem* testing programs. The performances of different TSE surveillance programs at population level were estimated based on the French goat population as an example, using a Monte Carlo simulation model. The model relied on the real structure and management practises of the French goat population and on the measured performances (table 2) of scrapie detection by post mortem PrP^Sc^ detection assays. The proportion of scrapie infected herds detected at abattoir (white boxes), rendering (black boxes) or in both streams together (grey boxes). In all cases PrP^Sc^ is carried out using ELISA (table 2). (A–B) 100% of animals eliminated from the commercial population are tested on posterior brainstem (A) or on palatine tonsil (B). (C–D) 10 000 animals are tested at abattoir and 10 000 animals are tested at rendering plant on either posterior brainstem (C) or palatine tonsil. (D).

To assess the performance of the TSE surveillance program at population level, using different scenarios, we set up a model using the French goat population (about 850 000 adult animals in 6 000 commercial herds) as an example (Figure 1).

In the first modelled scenario, we hypothesized that every goat aged over 2 years that was healthy slaughtered (128,000 animals) or eliminated at a rendering plant (50,000 animals) each year would be tested. In this scenario, the estimated proportion of infected herds that would be identified after one year of surveillance using post mortem PrP^Sc^ detection tests on posterior brainstem (“official rapid tests”) would be 51.03% (95% CI = 49.72–52.44) (Figure 1A). This proportion would reach 62.29% (95% CI = 60.94–63.76) if detection was carried out on palatine tonsil (Figure 1B).

In a second scenario, we considered that 20 000 tests would be randomly performed each year in fallen stock (10 000 animals) and healthy slaughtered (10 000 animals). In that scenario, after one testing year, PrP^Sc^ testing on posterior brainstem (“official rapid tests”), would result in the identification of 11.8% (95% CI = 10.7–12.7) of the scrapie infected herds (Figure 1C). If PrP^Sc^ detection should be carried out on palatine tonsils, 15.0% (95% CI = 13.9–16.1) of the infected herds would be identified (Figure 1D).

In both scenarios the capacity of the surveillance program to detect infected herds appeared to be strongly influenced by the herd size (Figure 1); the smaller the herd was, the lower were the detection performances. For instance, while scenario 1 was estimated to detect 75.1% (95% CI = 72.7–78.1) of infected herds that accounted more than 200 adults goats, only 30.5% (95% CI = 28.2–33.0) of the infected herds containing less than 50 adult goats could be detected.

## Discussion

Our study involved a relatively large number of classical scrapie cases (n = 183) recruited from eight naturally infected herds. It therefore provided a solid basis for assessing the performances of the TSE tests and/or choice of target tissue for classical scrapie detection in goats. However, like all previous studies focusing on the performance of PrP^Sc^ based TSE detection assay, this approach suffers from an intrinsic pitfall, *i.e*. the absence of a gold-standard that would provide a definitive TSE status for each individual. Currently, only bioassay (with no species barrier) could be considered as a pertinent tool to establish/exclude TSE agent presence in a sample. However, considering the number of samples that should be tested in an experiment like this one, systematic bioassay testing of each tissue is not feasible. In that context, we cannot exclude that some of the animals involved in this study might have been inaccurately considered to be TSE negative.

Since their implementation in the EU and in North America, the rationale of TSE active surveillance systems in small ruminants has been debated; the number and age of tested animals, the choice of TSE screening tests and the choice of testing on posterior brainstem have been extensively discussed [10]. As anticipated from knowledge related to classical scrapie pathogenesis in small ruminants (late neuro-invasion during the incubation phase), our results demonstrate that the detection of TSE infected individuals would be significantly improved by using tonsil or mesenteric lymph node rather than posterior brainstem for PrP^Sc^ testing [7], [18]. The high concordance of the results obtained using a rapid PrP^Sc^ detection assay (Biorad TeSeE sheep/goat®) and a reference OIE confirmatory method (IHC) indicates that there is no technical limitation for developing a field TSE surveillance program based on PrP^Sc^ testing in lymphoid tissues.

At the population level, whatever the testing scenario (number of tests performed each year) the results of our simulation study also indicated that TSE active surveillance strategies based on PrP^Sc^ testing on tonsil rather than on posterior brainstem would allow the detection of a higher number of classical scrapie infected herds. However, the performance gain (between 2% and 10% of additional scrapie infected herd detected) would remain relatively limited and whatever the testing regimen the system would fail to detect 100% of the infected herds.

In addition, it must be considered that in a large number of countries, small ruminant TSE surveillance programs intend to monitor the prevalence of both classical and atypical scrapie [6], [19]. The lack of detectable PrP^Sc^ in the peripheral tissue of atypical scrapie infected individuals precludes the use of lymphoid tissues for atypical scrapie surveillance [5].

Therefore, considering the current limits of TSE detection tests, and under the hypothesis that the sole objective of TSE surveillance programs is to provide a global picture of TSE prevalence in the small ruminants populations the use of posterior brainstem for PrP^Sc^ remains the best compromise for TSE monitoring in field.

Over the last decade, the EU policy for long term TSE control and eradication in small ruminants has relied on the identification of infected herds and the selection of genetically resistant animals in both infected flocks and in the general population [20]. In sheep, the ARR PRP allele provides a strong but not absolute resistance to classical scrapie and BSE agent [21], [22]. The recent identification of PrP polymorphisms in goats that might provide a strong, if not absolute, resistance to TSE agent infection could also provide opportunities for genetic selection in that species [23]–[26]. The selection of the ARR allele is an efficient tool for disease control/eradication in classical scrapie infected flocks [20]. However, the sustainability of genetic selection to control and eradicate TSE in small ruminants is still disputed. The existence of TSE agents (like atypical scrapie) that can develop in ARR homozygote sheep and the potential loss of genetic variability in animal populations are the two main arguments used by people arguing against genetic selection. Eradication policy based on the post mortem PrP^Sc^ testing and the stamping out of infected herds/flocks, without genetic selection is therefore still given consideration as an alternative to breeding for resistance. Our results clearly demonstrate that over a year the random testing of 20 000 individuals in an 850 000 individuals population only allow the identification of a very limited proportion (12%) of infected herds.

The simulation models that we developed rely on the French goat population and breeding system. They cannot be directly inferred to scrapie surveillance in sheep. However all the critical parameters that were used for modelling scrapie in the goat population were similar to those reported in sheep. The age distribution of cases that we used in goat was comparable to the one described in susceptible PrP genotype sheep [27], [28]. The age at which PrP^Sc^ become detectable in the posterior brainstem in goat (as reported here) and in naturally scrapie infected ARQ/ARQ sheep were also comparable [15]. In addition, the estimates obtained with our model are consistent with those reported by Hopp et al. [29] when assessing the performance of TSE active surveillance in the Norwegian sheep population. Therefore, it is our opinion that the results obtained in this study are also pertinent for estimating the likely performances of the post mortem scrapie surveillance program in sheep.

TSE in small ruminants is mainly considered as an animal health issue. However, the uncertainties related to the capacity of other ruminant prions to cross species barriers [30] remain a concern for public health. Considering the results of our study and the capacity of TSE agents to persist in the environment, it can be concluded that a classical scrapie eradication policy that would solely rely on currently available TSE screening tests is unlikely to succeed.

## Supporting Information

Figure S1 **Number of herds par size category (black bars) and number of female adult goats per herd size category (white bars) in the French goat population according to the AGRESTE 2007 database.** Only herds with 10 or more adult female goats were considered in the simulation study (n = 5928).
(TIF)

Figure S2 **Mean simulated age structure according to the high replacement rate (black bars) and the low replacement rate (white bars).**
(PDF)

Additional Text S1 **Description of the Monte-Carlo simulation model.**
(DOC)
