# Supplementary figures and images for: The Limits of Test-Based Scrapie Eradication Programs in Goats
Source: PLoS One. 2013 Jan 23;8(1):e54911. doi: 10.1371/journal.pone.0054911 (PMC3553010; doi:10.1371/journal.pone.0054911)

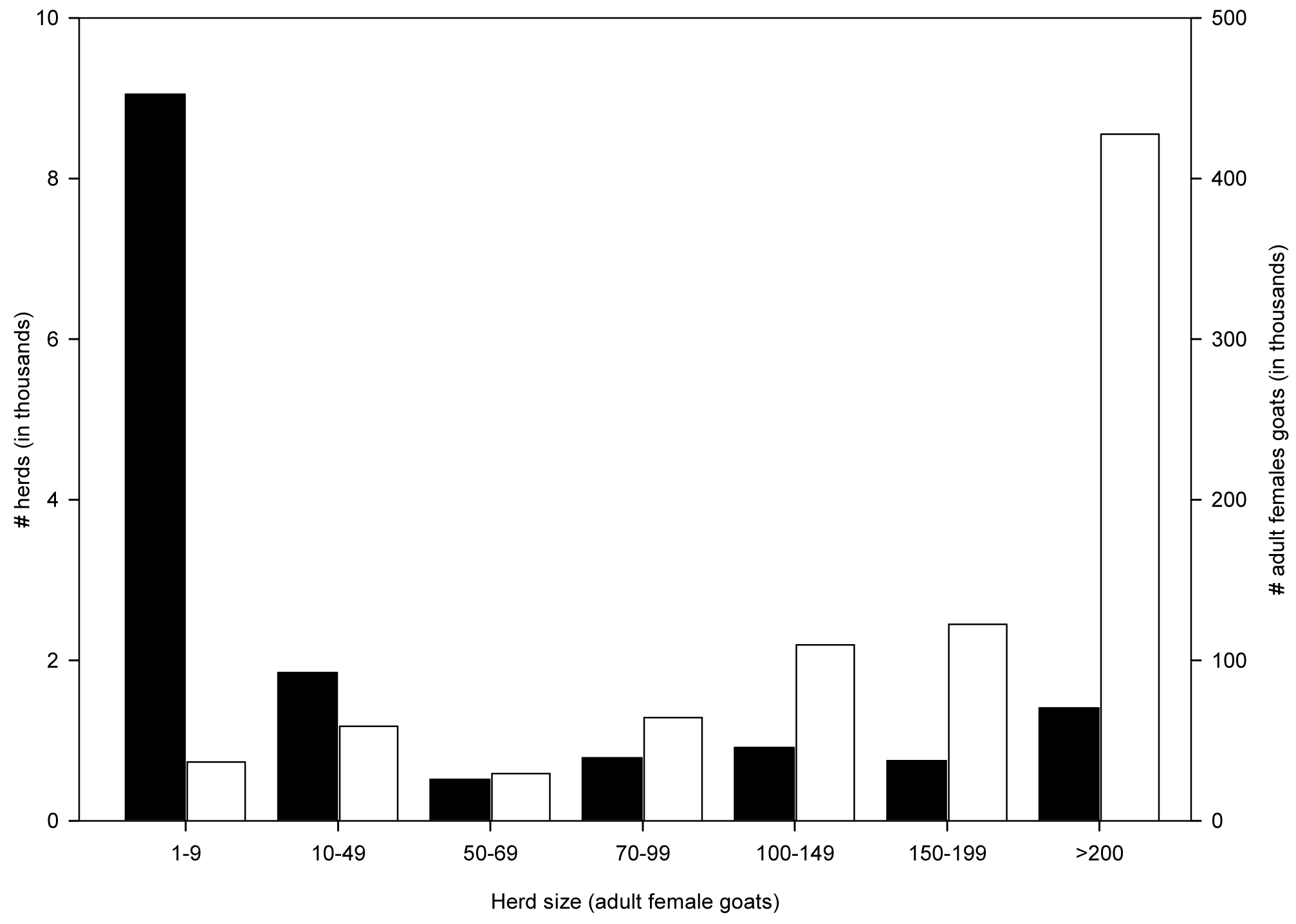

Supplement: Figure S1 — Number of herds par size category (black bars) and number of female adult goats per herd size category (white bars) in the French goat population according to the AGRESTE 2007 database. Only herds with 10 or more adult female goats were considered in the simulation study (n = 5928). (TIF) [file pone.0054911.s001.tif]

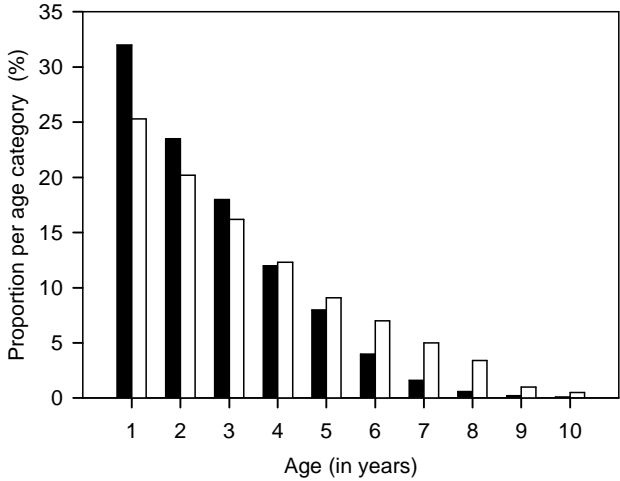

Supplement: Figure S2 — Mean simulated age structure according to the high replacement rate (black bars) and the low replacement rate (white bars). (PDF) [file pone.0054911.s002.pdf]
