# Supplementary material for: The Limits of Test-Based Scrapie Eradication Programs in Goats
Source: PLoS One. 2013 Jan 23;8(1):e54911. doi: 10.1371/journal.pone.0054911 (PMC3553010; doi:10.1371/journal.pone.0054911)
Supplement: Additional Text S1 — Description of the Monte-Carlo simulation model. (DOC) [file pone.0054911.s003.doc]

**Additional material to Corbière et al.: “The limits of test-based scrapie eradication programs in goats” - Description of the Monte Carlo simulation model.**

**French goat herds and population**

The data related to French herds were obtained from French ministry of agriculture records (AGRESTE 2007). In 2007, the population comprised 848 794 adult female goats in 15 268 herds. Amongst those 5938 had 10 or more adult female goats and represented more than 95.5% of the whole female goat population. Only herds with 10 or more adult goats were considered in our simulation study. The AGRESTE 2007 database only provided the number of herds within seven size categories (i.e. herd size 1-9, 10-49, 50-69, 70-99, 100-149, 150-199 and ≥200 goats) and the total number of adult female goats per size category (Figure S1). The simulations were based on the exact number of herds in each size category. For each herd within a size category, the herd size was randomly generated according to a uniform distribution, accept for size category ≥200 goats, for which a Pert(200, 230, 1000) distribution was used. Using these settings the difference between the simulated and the real number of adult female goats per herd size category was less than 5%.

Two type of age structures were randomly modelled according to available data . The first structure took into account a high renewal rate (mean age 3.6 years for goats older than 1 year) while the other corresponded to a lower renewal rate (mean age 4.3 years for goats older than 1 year). Each age structure was randomly assigned to the simulated herds. The age unit was set to one year and 10 groups from 1 year old to 10 years old goats were defined. Tabled probabilities, depending on the two age structures, were used to randomly assign the number of goats in each age group. Under these settings, the mean proportions of goats aged [1-2[, [2-5[ and [5-10] were 0.32, 0.54 and 0.14 for high replacement rate herds and 0.25, 0.49 and 0.26 for low replacement rate herds (Figure S2).

Within each herd, an age dependant replacement policy was modelled using tabled probabilities which allowed preservation of the age structure. Goats reaching 10 years old were considered to be systematically culled. The mean simulated culling rate was 29.3 % (95% CI = 21.9 – 44.4) for the high culling rate herd group design and 23.5 % (95% CI = 16.3 – 34.7) for the low culling rate herd group. The proportion of found-dead animals in adult goats (over 12 months old) was assumed to be independent of age and was modelled using a Pert distribution, with minimum 0, mode 7% and maximum 14%. Deaths from diseases other than Scrapie were considered to occur with equivalent frequency in Scrapie incubating and Scrapie free individuals.

**Scrapie incubation period**

The Scrapie incubation period (in years) was modelled using a Pert distribution with a minimal age at clinical onset of 1.5 years, mode of 3 years and maximum 12 years. The maximum incubation length selected was larger than the maximum lifespan to reflect the possibility that some infected and old animals may be eliminated because the end of their commercial lifespan preceded clinical onset. These parameters reflect the elements collected during the field study: amongst 33 clinical cases, the minimum, median and maximum age was 1.5, 3.2 and 8.7 years, respectively and some PrP^sc^ was detected in the obex of 4 clinically healthy goats older than 10 years.

Only a few clinical TSE goats cases were reported by the passive surveillance system in France (only one clinical suspicion case reported each year from 2006 to 2009, all un-confirmed) by comparison to the total number of cases reported by the global surveillance system. This observation is consistent with a limited efficiency of passive surveillance in goats. For convenience, clinically scrapie affected goats were therefore considered in our model to enter into the fallen stock rather than in a specific class of clinically suspect.

**Scrapie prevalence**

The distribution of the intra-herd classical scrapie prevalence in scrapie positive goat herds is unknown. Based on the results obtained in the eight studied herds, we generated a modified Pert distribution with minimum 1 infected animal, mode 5% and maximum 30%. Within an infected herd, the age distribution of scrapie infected goats was randomly assigned, to mimic the variability observed in the field which may be linked to the stochastic process of the epidemic, and to the fact that purchased animals may enter the herd and got infected at adult age.

**Performances of diagnostic tests**

The estimated sensitivities of the different tissues for the diagnosis of scrapie, obtained in the first phase of the study (investigation of field infected herds) were used. All tests were assumed to be 100% specific.

In goats incubating Scrapie the sensitivity of tests applied to obex was derived from a Pert (0.15; 0.25; 0.40) distribution for age 2 to 3 years and a Pert (0.30; 0.45; 0.60) distribution for goats of 3 and more years old. The sensitivity of diagnostic tests applied to tonsil was assumed to be independent of age and modelled through a Pert(0.80; 0.90; 0.98) distribution. These distributions closely reflect estimates and 95 % confidence intervals values obtained in field infected herds.

For animals having reached the end of their incubation period (either clinically affected, euthanized or found dead goats) the sensitivity of detection using obex and lymphoid tissues was assumed to be 100%.

**Model structure**

The simulation program consists of a sequence of difference equations. We assumed that (1) there is no selection measure to eliminate scrapie infected goat prior to the onset of clinical signs (2) goats are eliminated from each herd as a single batch each year.

Following Hopp et al. (2003) , a discrete time model was constructed using the following system of equations:

Number of clinical cases between age *a-1* and *a*

$$C(a)=I(a-1)\times h(a)$$

Number of found-dead animals between age *a-1* and *a*

$$D(a)=\left(N(a-1)-C(a)\right)\times r(a)$$

Number of culled animals between age *a-1* and *a*

$$Q(a)=\left(N(a-1)-C(a)-D(a)\right)\times q(a)$$

Number of infected goats at age *a*

$$I(a)=\left(I(a-1)-C(a)\right)\times\left(1-r(a)-\left(1-r(a)\right)\times q(a)\right)$$

Number of goats of age *a*

$$N(a)=N(a-1)-C(a)-D(a)-Q(a)$$

where *I(a-1)* is the number of infected animals of age *a-1*, *h(a)* the probability that an infected animal that is still alive just before age *a-1* will die between age *a-1* and *a*; *N(a-1)* is the total number of animals of age *a-1*; *r(a)* is the probability of an animal dying from other causes between age *a-1* and *a* and *q(a)* is the probability of an animal being slaughtered between age *a-1* and *a*.

**Active Surveillance scenarios**

Two scenarios were tested. In both scenarios, the minimum age of tested animals was assumed to be 2 years.

In the first scenario we considered that 100 % of slaughtered and found-dead goats older than 2 years would be tested, as it was the case in 2006 and 2007 in France.

In the second scenario we hypothesized that a limited number of tests were randomly performed at slaughter house and rendering plant (*n_1_* and *n_2_,* respectively). In this case the fact that an animal is tested or not is a Bernoulli trial with probability *p_1_ = n_1_/total number of slaughtered goats ≥ 2 years old* and *p_2_ = n_2_/total number of found-dead goats ≥ 2 years old* respectively.

In each simulation the number of iterations was set to 1,000. An infected goat herd was considered to be detected when at least one positive animal was recovered in that herd. In each surveillance scenario, the proportion of scrapie affected herds detected by the surveillance of slaughtered goats, fallen stock or using the combination of both fallen and slaughtered stock was recorded. Estimates and 95% confidence intervals were calculated using the median value and the 2.5% and 97.5% percentiles of the estimate distributions over the 1,000 iterations.

**Reference**

1. Malher X, Seegers H, Beaudeau F (2001) Culling and mortality in large dairy goat herds managed under intensive conditions in western France. Livestock Production Science 71: 75-86.

2. Hopp P, Webb CR, Jarp J (2003) Monte Carlo simulation of surveillance strategies for scrapie in Norwegian sheep. Prev Vet Med 61: 103-125.
